# Optimization of SARS-CoV-2 Spike Protein Expression in the Silkworm and Induction of Efficient Protective Immunity by Inoculation With Alum Adjuvants

**DOI:** 10.3389/fimmu.2021.803647

**Published:** 2022-01-12

**Authors:** Akitsu Masuda, Jae Man Lee, Takeshi Miyata, Hiroaki Mon, Keita Sato, Kosuke Oyama, Yasuteru Sakurai, Jiro Yasuda, Daisuke Takahashi, Tadashi Ueda, Yuri Kato, Motohiro Nishida, Noriko Karasaki, Kohei Kakino, Takeru Ebihara, Takumi Nagasato, Masato Hino, Ayaka Nakashima, Kengo Suzuki, Yoshino Tonooka, Miyu Tanaka, Takato Moriyama, Hirokazu Nakatake, Ryosuke Fujita, Takahiro Kusakabe

**Affiliations:** ^1^ Laboratory of Insect Genome Science, Graduate School of Bioresource and Bioenvironmental Sciences, Kyushu University, Fukuoka, Japan; ^2^ Laboratory of Creative Science for Insect Industries, Graduate School of Bioresource and Bioenvironmental Sciences, Kyushu University, Fukuoka, Japan; ^3^ Department of Biochemistry and Biotechnology, Faculty of Agriculture, Kagoshima University, Kagoshima, Japan; ^4^ Laboratory of Protein Structure, Function and Design, Faculty of Pharmaceutical Sciences, Kyushu University, Fukuoka, Japan; ^5^ Department of Emerging Infectious Diseases, Institute of Tropical Medicine, Nagasaki University, Nagasaki, Japan; ^6^ National Research Center for the Control and Prevention of Infectious Diseases, Nagasaki University, Nagasaki, Japan; ^7^ Graduate School of Pharmaceutical Sciences, Kyushu University, Fukuoka, Japan; ^8^ Laboratory of Sanitary Entomology, Graduate School of Bioresource and Bioenvironmental Sciences, Kyushu University, Fukuoka, Japan; ^9^ The Research and Development Department, Euglena Co., Ltd, Tokyo, Japan; ^10^ The Production Department, KAICO Ltd, Fukuoka, Japan

**Keywords:** SARS-CoV-2, spike (S) protein, silkworm-baculovirus expression vector system, COVID-19, adjuvant, paramylon, Alum

## Abstract

The newly emerged severe acute respiratory syndrome coronavirus 2 (SARS-CoV-2) is causing a spread of coronavirus disease 2019 (COVID-19) globally. In order to end the COVID-19 pandemic, an effective vaccine against SARS-CoV-2 must be produced at low cost and disseminated worldwide. The spike (S) protein of coronaviruses plays a pivotal role in the infection to host cells. Therefore, targeting the S protein is one of the most rational approaches in developing vaccines and therapeutic agents. In this study, we optimized the expression of secreted trimerized S protein of SARS-CoV-2 using a silkworm-baculovirus expression vector system and evaluated its immunogenicity in mice. The results showed that the S protein forming the trimeric structure was the most stable when the chicken cartilage matrix protein was used as the trimeric motif and could be purified in large amounts from the serum of silkworm larvae. The purified S protein efficiently induced antigen-specific antibodies in mouse serum without adjuvant, but its ability to induce neutralizing antibodies was low. After examining several adjuvants, the use of Alum adjuvant was the most effective in inducing strong neutralizing antibody induction. We also examined the adjuvant effect of paramylon from *Euglena gracilis* when administered with the S protein. Our results highlight the effectiveness and suitable construct design of the S protein produced in silkworms for the subunit vaccine development against SARS-CoV-2.

## Introduction

Severe acute respiratory syndrome coronavirus-2 (SARS-CoV-2) belongs to the genus *Betacoronavirus* in the family *Coronaviridae* and is genetically close to the 2003 outbreak of SARS-CoV and CoV isolated from bats (1, 2). SARS-CoV-2 is responsible for the symptoms called “Coronavirus disease 2019” (COVID-19), which causes a high fever and severe pneumonia in humans. The elderly, diabetics, or people with respiratory or cardiac disease are prone to severe disease (3). The COVID-19 cluster was initially discovered in a local seafood market in Wuhan, China. Despite the urban blockade, SARS-CoV-2 spread globally due to its high infectivity, causing a pandemic. As of July 19, 2021, the World Health Organization (WHO) announced 189,921,964 confirmed cases and 4,088,281 deaths globally by the spread of SARS-CoV-2.

Similar to other members of the CoV family, SARS-CoV-2 is an enveloped virus that uses spike (S) glycoprotein on the viral membrane to bind and enter the host cells. The receptor-binding domain of the S protein binds to the human angiotensin-converting enzyme 2 (ACE2), the same host receptor as SARS-CoV, and enters the host cell by membrane fusion (4). The S protein comprises two regions, S1 subunit and S2 subunit, forming a homotrimer (5). As well as many other CoVs, the S protein of SARS-CoV-2 is cleaved at the boundary between S1 and S2 by host proteases, such as the serine protease furin (6–10). The S1 subunit plays a pivotal role in attachment to the host cellular receptor, and the S2 subunit functions as membrane-fusion machinery (11).

So far, in addition to SARS-CoV-2, two highly pathogenic human CoVs, which are SARS-CoV and Middle East Respiratory Syndrome Coronavirus (MERS-CoV), and four relatively low pathogenic CoVs, which mainly cause the common cold in humans, have been discovered. However, no vaccine against coronaviruses is commercially available except for vaccines against SARS-CoV-2, which were developed and implemented at a phenomenal rate. The mRNA-based vaccines and recombinant virus-based vaccines that are already in use are designed to produce the S protein in the human body (12, 13). In addition, inactivated virus vaccines and recombinant protein vaccines have been developed, but the production cost of these vaccines is high, making it economically difficult to spread them throughout the world. Especially for subunit vaccines, both the baculovirus-insect cell and mammalian cell expression systems can produce S ectodomain at 5 mg/L, and the current efficiency is insufficient to produce inexpensive subunit vaccines, but the yield is expected to improve in the future (14, 15).

The silkworm-baculovirus expression vector system (BEVS) is frequently used to produce secreted proteins with complex higher-order structures (16). In particular, several reports have shown that recombinant antigens expressed using silkworms infected with Bombyx mori nucleopolyhedrovirus (BmNPV) are immunogenic against each pathogen (17–19). We have previously reported the successful production of recombinant S protein of SARS-CoV-2 in the trimeric state using this BmNPV-silkworm expression system (20). Our recent study also shows that fusion of coiled-coil derived from chicken cartilage matrix protein (CMP) is effective in stabilizing the ectodomain of porcine epidemic diarrhea virus (PEDV) S protein in the trimeric state and improving its secretion in silkworms (21). Based on the results of these studies, we have successfully improved the expression of the secreted product by optimizing the trimerization and purification tags. For the formulations of SARS-CoV-2 S protein-based vaccines, oil-in-water emulsion AS03, TLR9 agonist CpG, and the proven adjuvant Alum have been used as adjuvants in some immunological studies, and they have been shown to provide different immune effects (22, 23). Hence, selecting appropriate adjuvants is important to make the subunit vaccine fully effective, and in this study, we also examined several adjuvants suitable for the S antigen produced by silkworm-BEVS. Silkworm-derived S protein, which could be stably expressed by CMP fusion, elicited humoral immunity in immunized mice. In particular, the combination with Alum adjuvant prevented the infection of cultured cells with SARS-CoV-2 by efficiently inducing neutralizing antibodies. In addition, when mice were immunized with this silkworm-derived S protein using paramylon, a polysaccharide derived from *Euglena gracilis*, as a safer adjuvant, antibody induction against the S protein was observed. These mouse antisera significantly suppressed viral infection, although not as much as Alum adjuvant. These results demonstrate the efficient production of the S protein of SARS-CoV-2 in the silkworm, its immunogenicity as a subunit vaccine, and the effectiveness of the novel adjuvant in enhancing the immune effect.

## Materials and Methods

### Silkworm Cells and Strain

The BmN cell (Funakoshi Co., Ltd., Japan) was maintained in IPL41 insect medium (Sigma, St. Louis, MO) with 10% fetal bovine serum (FBS, Gibco, Grand Island, NY) at 27°C. The silkworm strains used in this study were provided by the Institute of Genetic Resources at Kyushu University, and the silkworm larvae were reared on mulberry leaves at 24–29°C.

### Construction of the pFastBac Expression Vectors

The expression plasmids were constructed in a single reaction through the Golden Gate assembly method (24). The *Bombyx mori* codon-optimized gene coding SARS-CoV-2 S glycoprotein ectodomain (1-1208 amino acid residues, NCBI Reference Sequence: NC_045512.2), three trimerization motifs, and four tag sequences with a protease cleavage site and a protein purification tag were synthesized and cloned into pUC57-Km (Genewiz). The original (not optimized) sequence of SARS-CoV-2 S glycoprotein ectodomain was amplified by PCR (20). The fragments were designed to possess unique overhangs after digestion with *Bsa*I. The S protein contains mutations in the furin cleavage site (682-GSAS-685) and proline substitutions (K986 and V987) to keep the S protein in a more stable state (5). Three trimerization motifs, bacteriophage T4 fibritin, CMP, and GCN4 were used to help S protein form a trimer. Four types of tags that combine three sequences (TEV or HRV3C and His6 or His8, and STREP or TwinSTREP) were used for a protease cleavage site and a protein purification tag. For the Golden Gate cloning system, the commercially available pFastBac vector was modified into pFastBac L21-GG, in which the *ccd*B cassette flanked by two *Bsa*I sites with the unique overhangs was inserted in the multiple cloning site, and two native *Bsa*I sites were removed. Equimolar amounts of the indicated trimerization motif and tag plasmids, the spike gene plasmid, and pFastBac L21-GG were mixed, and the Golden Gate reactions were performed with T4 DNA ligase and *Bsa*I-HFv2 in 1X T4 DNA ligase buffer (NEB) for 15 cycles of 5 min at 37°C and 10 min at 16°C. Then, 1 μL of the reaction was transformed into *E. coli* DH10B. All the constructed plasmids were confirmed by Sanger sequencing.

The pFastBac-SARS2/SNF+knob+TEV-H8STREPH6 vector, which contains SARS-CoV-2 S glycoprotein ectodomain (not codon-optimized) with a mutation in the furin cleavage site (682-GSAS-685) and fused with the β-sheet domain of CELO long fiber knob (aa 583-590), was constructed previously (20).

### Generation of Recombinant Baculovirus

The resulting pFastBac plasmids were transformed into *E. coli* DH10Bac cells to generate the recombinant bacmids (Qd04 strain) of BmNPV (20, 25). Each recombinant bacmid DNA was transfected into BmN cells using 1:1:1 transfection reagent (dioleoylphosphatidylchorine: dioleoylphosphatidylethanolamine:polyethylenimine 1800Da at a 1:1:1 ratio) according to previous report (26). Briefly, BmN cells seeded on 24-well plates in IPL41 medium supplemented with 10% FBS were replaced with serum-free KBM720 medium (Kohjin Bio, Japan) before transfection. Then, recombinant bacmid DNA dissolved in HEPES-buffered saline was mixed with 1:1:1 transfection reagent and added to the BmN cells in KBM720 medium. After incubation at 27°C for 12h, the medium was replaced with IPL41 medium supplemented with 10% FBS and incubated at 27°C for 4 days. The high titer stocks were obtained by serial infections and kept dark at 4°C until use. The plaque assay determined the titers of each recombinant virus.

### Expression Analysis of Recombinant SARS-CoV-2 Spike Protein in Silkworm

On the third day of the 5th instar, silkworm larvae were injected with each recombinant BmNPVs (1 × 10^4^ PFU per larva). At 1-4 and 5 days post-infection (dpi), the sera of infected larvae were collected by cutting the proleg and centrifuging at 1,000 g for 10 min at 4°C.

All samples were mixed with two-fold SDS sample buffer (0.1 M Tris-HCl pH 6.8, 0.2 M dithiothreitol, 4% SDS, 20% glycerol, and 0.02% bromophenol blue) and denatured at 96°C for 10 min for SDS-PAGE and Western blotting analysis.

### Screening of Suitable Silkworm Strains for Efficient Production of Recombinant SARS-CoV-2 Spike Protein

Thirteen silkworm strains were employed for screening analysis. Each fifth-instar silkworm larva (day 3) was infected with the recombinant BmNPV, which expresses SARS2/SNFPP+CMP+TEV-H8STREPH6 at 1 × 10^4^ PFU per larva. At the 4 dpi, the sera of each silkworm were collected on ice separately. After centrifugation at 3,000 g for 5min, the supernatants were collected and stocked at -80°C until use.

All samples were mixed with SDS sample buffer (0.1 M Tris-HCl pH 6.8, 0.2 M dithiothreitol, 4% SDS, 20% glycerol, and 0.02% bromophenol blue) and denatured at 96°C for 10 min. The samples were subjected to Western blotting analysis (Described in SDS-PAGE and Western blotting section). The S proteins were detected using HisProbe-HRP (Thermo Fisher Scientific, USA). The intensities of each specific band were measured using ImageJ software version 1.51s, and the relative intensities were calculated by setting the mean intensity of the f38 strain to 1.

### Purification of Recombinant SARS-CoV-2 Spike Protein From Silkworm Serum

At 4 days after infection, the 10 mL serum was collected from infected silkworm larvae and centrifuged at 1,000 g for 10 min at 4°C. The serum supernatant was up to 50 mL with 1 × T buffer (20mM Tris-HCl, 0.5 M NaCl, pH 7.5), and 25 mL of each sample was sonicated for 10 min by TOMY UD-100 (Tomy Seiko Tokyo, Japan) at 4°C. After further centrifugation at 12,110 g for 30 min at 4°C, the supernatant was filtrated by 0.45 µm filters (Millipore, USA). The filtered sample was loaded onto a 5 mL HisTrap excel column (GE Healthcare Life Sciences, England) equilibrated with 1 × T buffer containing 20 mM imidazole. After washing the column with 1 × T buffer containing 30 mM imidazole (25 mL), the elution step was performed using 1 × T buffer containing 500 mM imidazole (25 mL). Next, the buffer of the eluent of nickel affinity chromatography was exchanged to 1 × PBS (137 mM NaCl, 2.7 mM KCl, 10 mM Na_2_HPO_4_·12H_2_O, 1.8 mM KH_2_PO_4_, pH 7.4) by ultrafiltration, and then the sample was applied to a 5 mL Strep-Tactin Superflow column (50 mL) (IBA GmbH, Germany). After washing the column with 1 × PBS (25 mL), the S proteins were eluted with 1 × PBS supplemented with 2.5 mM desthiobiotin (25 mL). The elution fractions were concentrated by ultrafiltration using Amicon Ultra-15 100K filters (Millipore, USA). Finally, the S protein was dialyzed in 1 × PBS and stocked at -80°C until use.

The purified S protein was quantified using the DC protein assay kit (BioRad, USA) according to the manufacturer’s protocol using BSA as a standard.

### SDS-PAGE and Western Blotting

All protein samples for SDS-PAGE analysis were mixed with an equal volume of two-fold SDS sample buffer (0.1 M Tris-HCl pH 6.8, 0.2 M dithiothreitol, 4% SDS, 20% glycerol, and 0.02% bromophenol blue) and denatured at 96°C for 10 min. The samples were separated by 8% SDS-PAGE and stained by Coomassie Brilliant Blue (CBB). For SDS-PAGE under non-reducing conditions, samples were mixed with two-fold SDS buffer without dithiothreitol (0.1 M Tris-HCl pH 6.8, 4% SDS, 20% glycerol, and 0.3% orange G).

The proteins separated by SDS-PAGE were transferred to PVDF membrane (Millipore, Milford, MA) and further blocked with Tris-buffered saline with 0.1% Tween 20 (TBST) containing 5% skim milk (Wako, Tokyo, Japan). After being washed 5 times with TBST, the membrane was incubated with mouse monoclonal antibody against SARS-CoV-2 Spike RBD (ab277624, Abcam, Cambridge, UK) for 1h at 37°C and anti-mouse IgG conjugated with peroxidase (Sigma–Aldrich, St. Louis, MO) for 90 min at 37°C. The specific protein bands were visualized using the Super Signal West Pico Chemiluminescent Substrate (Thermo Fisher Scientific, USA).

HisProbe-HRP (﻿Thermo Fisher Scientific, USA) was also used to detect His-tagged protein in the Western blotting analysis.

### Blue-Native PAGE

According to the manufacturer’s instructions, purified S proteins were mixed with NativePAGE 4 × sample buffer and electrophoresed on NativePAGE 3-12% bis-tris gel (Thermo Fisher Scientific, USA). Samples were separated at 150 V for 115 min in NativePAGE running buffer (Thermo Fisher Scientific, USA) containing NativePAGE cathode buffer additive (Thermo Fisher Scientific, USA). Only the Native PAGE running buffer was used as anode buffer. The bands of proteins were visualized by staining gels with CBB.

### Size-Exclusion Chromatography

Purified SARS2/SNFPP+CMP+TEV-H8STREPH6 was subjected to size-exclusion chromatography using a superose 6 increase 10/300 GL column (Cytiva, Tokyo, Japan) in 1 × PBS buffer. The peak fractions were collected and concentrated by ultrafiltration using Amicon ultra-15 100K filters (Merck, USA). Thyroglobulin (669 kDa), Ferritin (440 kDa), and Aldolase (158 kDa) were used as molecular weight markers.

### Deglycosylation Assay

PNGase F (27), O-glycosidase (NEB, USA), and Endo H (28) digestion reactions were performed according to the manufacturer’s instructions. In brief, ﻿recombinant SARS-CoV-2/SNFPP+CMP+TEV-H8STREPH6 was mixed with the denaturing buffer (NEB) and denatured at 96°C for 10 min. Subsequently, the denatured proteins were deglycosylated by PNGase F, O-glycosidase, or Endo H at 37°C for 3h. The reactions of denatured samples were stopped by mixing with two-fold SDS sample buffer and heating for 10min at 96°C. For Blue-Native PAGE, the enzymatic reaction was performed on the samples that were not heat-denatured. All samples were analyzed by SDS-PAGE and Blue-Native PAGE.

### ACE2 Binding Assay

Recombinant human ACE2 proteins (R&D) were coated to Nunc maxisorp 96-well plates (Thermo Fisher Scientific, USA) at the concentration of 1 µg/mL, followed by incubation at 4°C overnight. The plates were washed 3 times with 1 × PBS containing 0.1% Tween 20 (PBST) and blocked with 5% skim milk (Wako, Tokyo, Japan) in PBST for 1h at room temperature. After being washed with PBST, the plates were added serial dilutions of the SARS2/SNFPP+CMP+TEV-H8STREPH6 proteins and incubated for 1h at room temperature, then washed 4 times with PBST. STREP tactin-HRP (IBA GmbH, Germany) was added to wells. After incubation for 1h at room temperature, the plates were washed 4 times with PBST and developed with 3,3’,5,5’-tetramethylbiphenyldiamine (TMB, Merck, USA). The reactions were stopped with 2M H_2_SO_4_, and the absorbance at 450 nm wavelength was measured by a Synergy HTX plate reader (BioTek Instruments, Highland Park, VT).

### Immunization of Mice

The mice experiments in **Figure 5** were conducted according to relevant national and international guidelines ’Act on Welfare and Management of Animals’ (Ministry of Environment of Japan) and ’Regulation of Laboratory Animals’ (Kyushu University), and under the protocols approved by the Institutional Animal Care and Use Committee review panels at Kyushu University (Approval number: A20-241-0).

In the mice experiments in **Figure 6**, the mice were maintained and the experiments were performed according to the approved guidelines from Animal Care and Use Committee of Kagoshima University (Approval number: A19012).

Seven-week-old female BALB/c mice were injected intraperitoneally 3 times with 0.12, 1.2, or 12 µg of SARS2/SNFPP+CMP+TEV-H8STREPH6 proteins 14-day intervals with or without various adjuvants (Freund’s Complete Adjuvant (CFA, Difco laboratories, NJ, USA), Freund’s Incomplete Adjuvant (IFA, Difco laboratories) aluminum hydroxide (Imject Alum, Thermo Fisher scientific, USA) and paramylon (Euglena, Japan), respectively. Paramylon is a polysaccharide contained by the microalga *Euglena gracilis*, and was provided by euglena Co., Ltd. Paramylon was isolated as described previously (29). The serum of each immunized mice was collected at 42 days post-immunization, respectively.

### Antigen Binding ELISA

**Figure 1:**
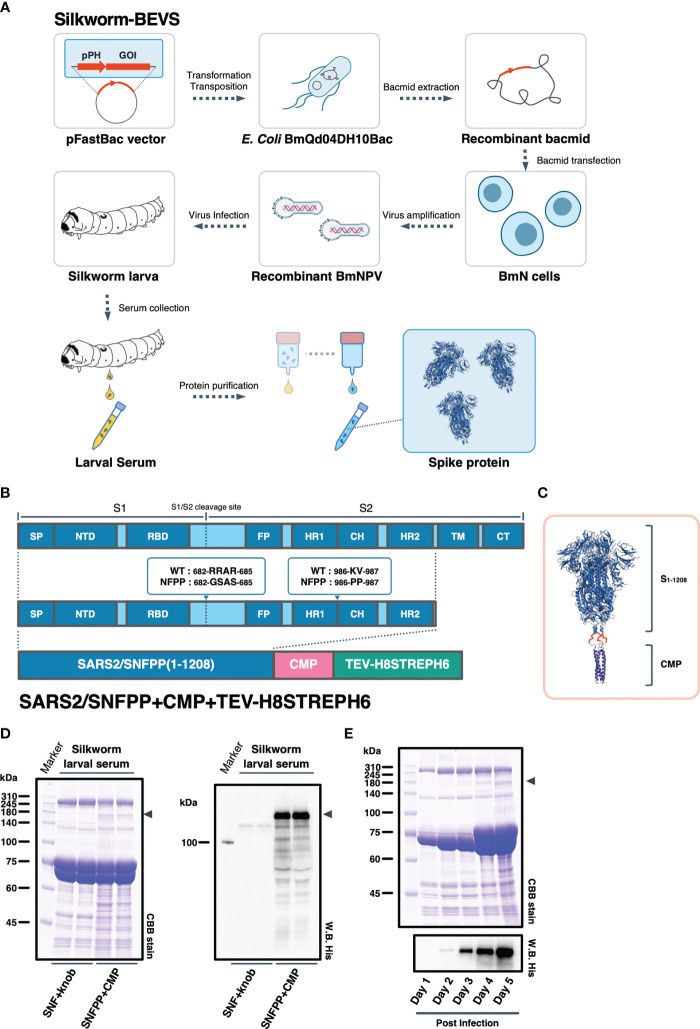
Expression constructs and expression analysis of SARS-CoV-2 spike protein. **(A)** Schematic representation to produce recombinant SARS-CoV-2 spike protein using silkworm-BEVS. **(B)** A map of the domains of the spike protein. SP, signal peptide; NTD, N-terminal domain; RBD, receptor binding domain; FP, fusion peptide; HR1, heptad repeat 1; CH, central helix; HR2, heptad repeat 2; TM, transmembrane domain; CT, cytoplasmic tail. The expression construct of SARS2/SNFPP+CMP+TEV-H8STREPH6 using chicken cartilage matrix protein (CMP) and the Tags contains a tobacco etch virus protease cleavage site, 8×histidine-tag (H8), STREP(II)-tag, and 6×histidine-tag (H6). **(C)** Schematic representation of the trimeric SARS-CoV-2 spike protein (PDB:6vxx) fused with CMP (PDB:1aq5). **(D)** Expression of SARS-CoV-2 spike protein in silkworm larva. The sera of silkworm larvae expressing SNF+knob or SNFPP+CMP were collected at 4 days after recombinant BmNPV infection. All samples (sera of 1µL) were analyzed by SDS-PAGE followed by CBB staining (left panel) and Western blotting using HisProbe-HRP (right panel). Arrowhead indicates the position of the S protein. Molecular sizes (kDa) of the protein markers are indicated on the left. **(E)** Time course analysis of the expression of SARS2/SNFPP+CMP+TEV-H8STREPH6 in the serum of silkworm larvae. The sera were collected at the indicated days after recombinant BmNPV infection. Samples (sera of 1µL) were analyzed by SDS-PAGE followed by CBB staining or Western blotting with HisProbe HRP. Molecular sizes (kDa) of the protein marker are indicated on the left.

The 96-well ELISA plates (Sumilon type S, Sumitomobakelight co., ltd, Japan) were coated with 50 or 100 μL of 1 μg/mL of the SARS2/SNFPP+CMP+TEV-H8STREPH6 proteins or SUMO-SARS2/RBD+TEV-H8STREPH6 proteins in bicarbonate buffer (pH9.6) and incubated overnight at 4°C. The plates were washed with PBS containing 0.1% Tween-20 (PBST) and PBS and blocked with a blocking buffer (PBS containing 1% BSA) at room temperature for 1 h. After washing, 50 μL of each diluted serum sample was added to each well. Next, the plates were washed, and 50 μL of 0.5% BSA containing a peroxidase-conjugated goat anti-mouse IgG Fab antibody (Jackson immunoresearch, Laboratories, Inc., West Grove, PA, USA) was added into each well and incubated for 1 h. Finally, the plates were washed with PBST and PBS, and the color reaction was developed with ABTS solution (**Figures 5B, D**) or 1-Step Turbo TMB-ELISA substrate solution (**Figures 6B, C**, Thermo Fisher Scientific, USA). The optical density at 405 nm (OD405nm) or 450 nm (OD450nm) was read using a microplate reader.

### SARS-CoV-2 Neutralization Assay

A neutralization assay using wild-type SARS-CoV-2 was performed using VeroE6 cells constitutively expressing human TMPRSS2 (VeroE6/TMPRSS2 cells), provided by National Geographic Institutes of Biomedical Innovation, Health and Nutrition (NIBIOHN), Osaka, Japan. For infection, a JPN/NGS/IA-1/2020 strain of SARS-CoV-2 (accession number: EPI-ISL-481251, GISAID) was used after propagation in VeroE6 cells using Dulbecco’s modified Eagle medium (DMEM; Sigma Aldrich, St. Louis, MO, USA) supplemented with 10% fetal bovine serum (FBS) and 1% penicillin/streptomycin solution. The day before infection, VeroE6/TMPRSS2 cells were seeded in 96-well plates to reach 100% confluence and cultured in DMEM supplemented with 2% FBS at 37°C. Each mouse serum was inactivated by heating at 56°C for 30 minutes, filtrated and then diluted 50-fold in DMEM. SARS-CoV-2 (2.0×10^3^ pfu/mL) and an equal volume of diluted mice serum were mixed and incubated for 1h at 37°C. The mixtures were diluted serial 10-fold and transferred to the 96 well plates containing VeroE6/TMPRSS2 cells. After 3 days of incubation at 37°C, the cytopathic effects of each well were observed under a microscope and calculated median tissue culture infectious dose (TCID_50_). As positive controls, a COVID-19 patient serum was used for the 1st round experiment (**Figure 5E**), and the sample of 30 µg S + Alum in the 1st round experiment was used for the 2nd round experiment (**Figure 6D**). All experiments with replication-competent SARS-CoV-2 were performed in a biosafety level 3 laboratory at Nagasaki University.

### Statistical Analysis

Results of the SARS-CoV-2 neutralization assay were analyzed using an unpaired t-test to determine statistical significance compared to the mock-treated sample (Negative control). A difference of P < 0.05 was considered to be significant. The analyses were performed using GraphPad Prism software (GraphPad Software, San Diego, CA, USA).

## Results

### Construction and Expression of the SARS-CoV-2 Spike Protein

We have successfully expressed S proteins of several coronaviruses using silkworm-BEVS (21), and in this study, we attempted to produce S proteins of SARS-CoV-2 more efficiently and at a lower cost. The schematic diagram of recombinant protein production in silkworm-BEVS is briefly shown in **Figure 1A**. Using this method and based on our previous report of porcine epidemic diarrhea virus S protein expression in silkworm using CMP as a trimerization domain (21), we generated a construct named SARS2/SNFPP+CMP+TEV-H8STREPH6 to express the S protein of SARS-CoV-2 efficiently. In this construct, three amino acid substitutions were made in the furin recognition site (residues 682-685) of the ectodomain (1-1208aa) of the SARS-CoV-2 S protein to make it resistant to cleavage, and two proline mutations (residues 986 and 987) were introduced to stabilize the conformation. In addition, a natural trimeric chicken cartilage matrix protein (CMP) was fused *via* a peptide linker (red linkers in **Figure 1C**) to the C-terminus as a trimeric motif (**Figure 1B**). For efficient purification, the C-terminal region of CMP was tandemly fused with the cleavage site of tobacco etch virus protease, 8× histidine tag, STREP(II) tag, and 6× histidine tag (**Figure 1B**).　The expression in silkworm larvae was confirmed by SDS-PAGE followed by CBB staining and Western blotting with His-probe, which showed that, compared to the previously reported SARS2/SNF+knob+TEV-H8STREPH6 (20), the introduction of the proline mutation and fusion of CMP greatly improved the secretory expression in silkworm serum (**Figure 1D**). In mammalian cells, the addition of four more proline substitutions (SNFPP+4P) has been reported to improve the expression (30), whereas the expression of SNFPP+4P was comparable to that of SNFPP in silkworm serum (**Supplementary Figures 1A, B**). Time-course analysis of SARS2/SNFPP+CMP+TEV-H8STREPH6 expression in silkworm larvae after baculovirus infection showed that S protein in serum was highest at 5 days post-infection (dpi) (**Figure 1E**), but high mortality was observed at 5 dpi (data not shown).

### Identification of Suitable Silkworm Strains for Production of the Recombinant SARS-CoV-2 Spike Protein

**Figure 2:**
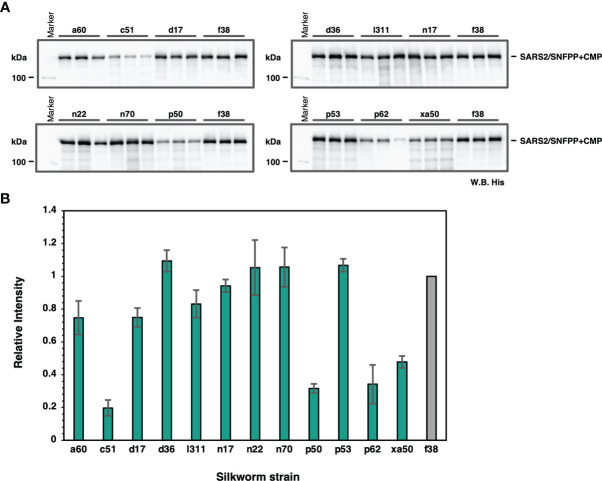
Screening of suitable silkworm strains for efficient SARS-CoV-2 S protein expression. **(A)** The Western blotting analysis of 13 silkworm strains infected with recombinant baculovirus encoding SARS2/SNFPP+CMP+TEV-H8STREPH6. The proteins in the sera of 0.5 μL were separated by 8% SDS-PAGE, and the specific band was detected using HisProbe-HRP. Three individual samples were prepared from each strain, and f38 strain was used as a control. **(B)** Relative band intensities of SARS-CoV-2 S protein obtained by Western blotting of sera from different silkworm strains. The intensities of each band were measured by ImageJ software, and the relative intensities were calculated using the average band intensity of f38 strain as 1. The data are represented as mean ± SEM (n = 3).

To find suitable silkworm strains for efficient production of SARS-CoV-2 spike protein, we performed a comparative analysis of secretory expression levels using 13 inbred silkworm bioresource strains maintained at Kyushu University. Sera of silkworm larvae inoculated with recombinant baculoviruses were collected at 4 dpi and used for Western blotting analysis to compare the expression levels among strains, and the expression levels of SARS2/SNFPP+CMP+TEV-H8STREPH6 protein in sera differed substantially among strains (**Figure 2A**). In particular, d36, f38, n22, n70, and p53 strains showed high expression levels (**Figure 2B**).

### Optimization of S-Protein Secretory Expression of SARS-CoV-2 by a Combination of Trimerization Motifs, Protease Cleavage Sites, and Affinity Tags

**Figure 3:**
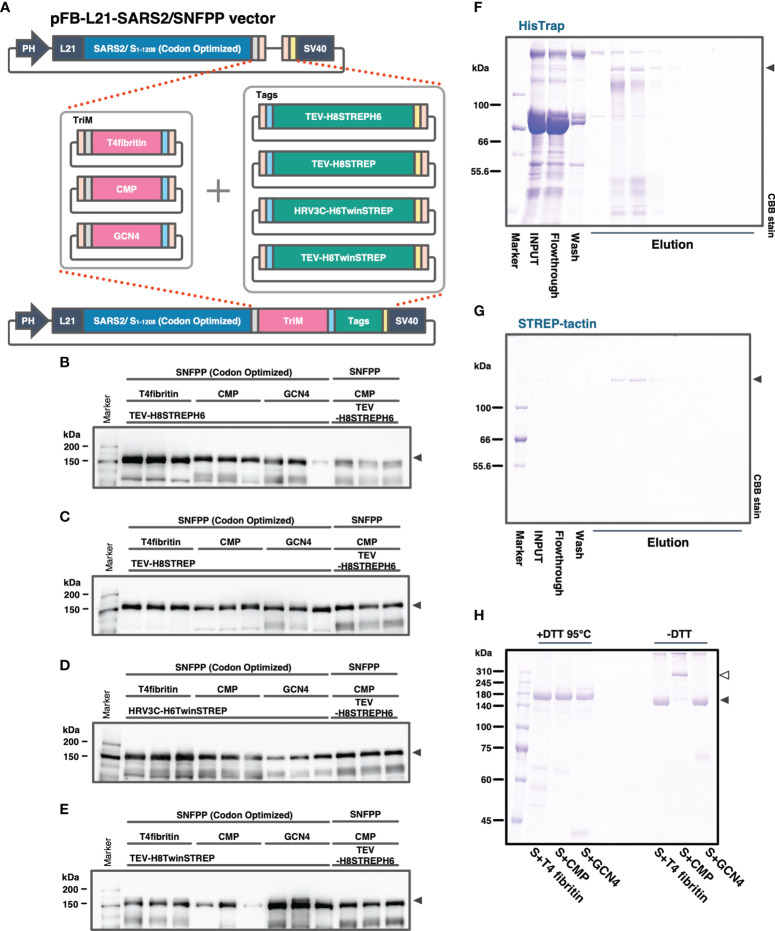
Comparison of expression constructs for optimization of SARS-CoV-2 S protein expression in silkworm-BEVS. **(A)** Schematic diagram of the construction of expression vectors for the S proteins with different combinations of trimerization motifs, protease cleavage sites, and affinity tags by the golden gate cloning method. SARS-CoV-2 SNFPP(1-1208) was codon-optimized for *Bombyx mori*. TriM: trimerization motifs (T4 fibritin, CMP, or GCN4), Tags: The combinations of protease cleavage sites and affinity tags (TEV-H8STREPH6, TEV-H8STREP, HRV3C-H6TwinSTREP, or TEV-H8TwinSTREP). **(B–E)** The Western blotting analysis of 12 expression constructs. The proteins in the sera of 0.5 μL were separated by 8% SDS-PAGE, and the specific band was detected using mouse monoclonal antibody against SARS-CoV-2 S receptor binding domain. Three individual samples were prepared from each construct, and SARS2/SNFPP+CMP+TEV-H8STREPH6 (without codon-optimized) was used as a standard. **(F, G)** Purification of SARS-CoV-2/SNFPP+CMP+TEV-H8STREPH6 (with codon-optimized) from silkworm larval serum (n70 strain) using the two-step affinity chromatography. All samples (10µL) from each step of the purification process were separated by 8% SDS-PAGE and visualized by CBB staining. **(H)** SDS-PAGE analysis of purified SARS-CoV-2/SNFPP+T4fibritin+TEV-H8STREPH6, SARS-CoV-2/SNFPP+CMP+TEV-H8STREPH6, or SARS-CoV-2/SNFPP+GCN4+TEV-H8STREPH6 protein under reducing or non-reducing conditions. The monomer and oligomer of the S protein were indicated by the black and white arrowhead, respectively.

In our previous study, the SARS2/SNFPP+CMP+TEV-H8STREPH6 recombinant baculovirus was constructed using the original SARS-CoV-2 S gene sequence. In subsequent studies, we used the sequence optimized for *Bombyx mori* codon usage and optimized the secretory expression by combining trimerization motifs, protease cleavage sites, and affinity tags. At the same time, we also examined the effect of codon optimization. To efficiently compare the effects of the three factors, a seamless cloning method, Golden Gate assembly, was used to add three trimerization motifs (T4fibritin, CMP, GCN4), four combinations of protease cleavage sites and affinity tags (TEV-H8STREPH6, TEV-H8STREP, HRV3C-H8TwinSTREP, TEV-H8TwinSTREP), which resulted in a total of 12 expression constructs were constructed (**Figure 3A**). Then, we infected silkworm larvae with recombinant baculoviruses produced from these expression vectors and compared the S protein expression levels by Western blotting of sera at 4 dpi with antibody against receptor binding domain (RBD) (**Figures 3B–E**). Among all the expression constructs, the most enhanced expression of S-protein was observed in the construct fusing T4fibritin with TEV-H8STREPH6 (**Figures 3B–E**). The effect of protease cleavage sequences and affinity tags showed a similar trend independent of the choice of trimerization motif. Focusing on the trimerization motifs, the expression level of T4fibritin was generally high, followed by CMP (**Figure 3B**). Codon optimization was also very effective in the efficient secretory expression of SARS2/SNFPP. Exceptionally, the expression of two constructs fusing GCN4 with HRV3C-H6TwinSTREP and CMP with TEV-H8TwinSTREP was reduced by codon optimization (**Figures 3D, E**).

Next, we attempted to purify the S proteins from 10 mL of larval serum of the n70 strain by two-step affinity chromatography against His-tag and STREP-tag. By a two-step purification, the SARS2/SNFPP+CMP+TEV-H8STREPH6 protein was successfully isolated from a very miscellaneous protein in silkworm serum, with a final yield of about 215 μg from 10 mL of serum (**Figures 3F, G**). Although the construct using T4fibritin as the trimerization domain had the highest expression level, the amount recovered after purification was not as high as expected, only approximately 1.49 times higher than that of the construct using CMP (data not shown). In addition, unlike T4fibritin and GCN4, there are multiple intermolecular disulfide bonds at the N-terminus of the CMP trimerization domain, which are expected to stabilize the trimer of the S protein. Therefore, SDS-PAGE was performed under reducing and non-reducing conditions, and only the band of the S protein fused with CMP was shifted upward in the non-reducing condition compared with that fused with T4fibritin and GCN4, suggesting stable oligomer formation by disulfide bonds (**Figure 3H**). From these results, for further characterization and immunization experiments, we decided to use a construct using CMP trimerization domain, which had been shown to have a high ability to induce neutralizing antibodies in vaccine production against PEDV (21).

### Characterization of the Recombinant SARS-CoV-2 Spike Protein

**Figure 4:**
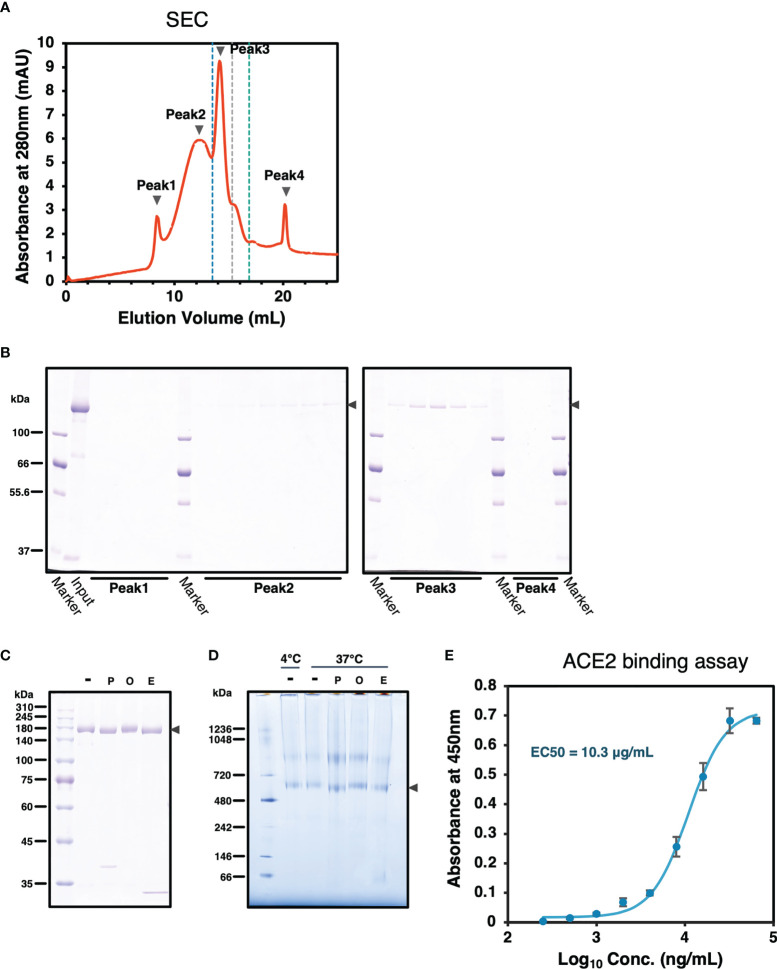
Characterization of SARS-CoV-2 S protein. **(A)** A chromatograph of the spike protein on a superose 6 increase 10/300 GL column. The black arrowheads indicate peaks 1 to 4. The blue, grey, and green dashed lines indicate the molecular weight markers of 669 kDa, 440 kDa, and 158 kDa, respectively. **(B)** Samples from each peak fraction of size-exclusion chromatography were separated by 8% SDS-PAGE. The black arrowheads indicate the S protein. **(C)** Deglycosylation assay of the SARS-CoV-2 S protein from the silkworm. -: no treatment, P, PNGase F; O, O-glycosidase; E, Endo H. The black arrowhead indicates the S protein. **(D)** Blue-Native PAGE analysis of the SARS-CoV-2/SNFPP+CMP+TEV-H8STREPH6 protein. All samples, including from deglycosylation assay without denaturing, were separated by 3-12% Bis-Tris gradient gel and stained by CBB. -: no treatment, P, PNGase F; O, O-glycosidase; E, Endo H. The black arrowhead indicates the S protein trimer. **(E)** Measurement of the spike protein binding to ACE2. Human ACE2 protein at the concentration of 1 µg/mL was coated on a 96 well plate, evaluating the binding ability of serial dilutions of SARS-CoV-2 spike protein. The data represented as mean ± SEM (n = 3).

To confirm SARS-CoV-2 S protein forms trimer, size-exclusion chromatography (SEC) was performed followed by SDS-PAGE analysis. SARS2/SNFPP+CMP+TEV-H8STREPH6 protein was eluted in peaks 2 and 3 (**Figures 4A, B**). The peak 3 appearing between the molecular weight standards of 440 kDa and 669 kDa corresponds to the trimerized S protein, and peak2 is expected to be a larger complex (**Figure 4A**). These results showed that most SARS2/SNFPP+CMP+TEV-H8STREPH6 proteins purified from silkworm serum formed trimeric or larger complexes, and few molecules were found to exist as monomers.

From the site-specific mass spectrometry analysis, it has been reported that the S protein protomer produced in HEK293 cells of mammalian origin has 22 N-glycans, most of which are of the complex type (31). Since it has been reported that paucimannosidic-type N-glycans are attached to the proteins expressed and secreted in insect cells (32), the glycosylation of the SARS2/SNFPP+CMP+TEV-H8STREPH6 protein produced by this expression system was analyzed. The deglycosylation of S protein using N-glycosidase F (PNGase F), O-glycosidase, and endoglycosidase H (Endo H) showed a decrease in molecular weight by PNGase F or Endo H treatment (**Figure 4C**). Consistent with this result, BN-PAGE results showed that the observed bands of the S trimer were shifted downward by PNGase F or Endo H treatment to remove the glycans (**Figure 4D**). PNGase F cleaves almost all types of N-glycans attached to asparagine residues of proteins, whereas Endo H cleaves only high-mannose and certain types of hybrid glycans. High-mannose and paucimannose type N-glycan structures are predominantly found in glycoproteins of butterflies, moths, and flies (33). These results are consistent with those of protein obtained in the insect cell-baculovirus expression vector system using cultured *Trichoplusia ni* cells (14), suggesting that in lepidopteran insects including silkworm, the S protein expressed as a recombinant protein is modified mainly by high-mannose-type N-glycosylation. Similar to the results of SEC analysis, BN-PAGE results also showed that the band observed above the 720 kDa molecular weight marker was predicted to form a larger complex than the trimer (**Figure 4D**).

The binding ability of recombinant SARS-CoV-2 spike proteins to the receptor was examined by human angiotensin-converting enzyme 2 (hACE2) biding assay. The purified SARS2/SNFPP+CMP+TEV-H8STREPH6 proteins bound to hACE2 in a dose-dependent manner (**Figure 4E**). These results indicated that SARS2/SNFPP+CMP+TEV-H8STREPH6 expressed in silkworms can act as a receptor binding machinery similar to SARS-CoV-2 spike protein.

### Humoral Immune Responses in the S Protein Immunized Mice

**Figure 5:**
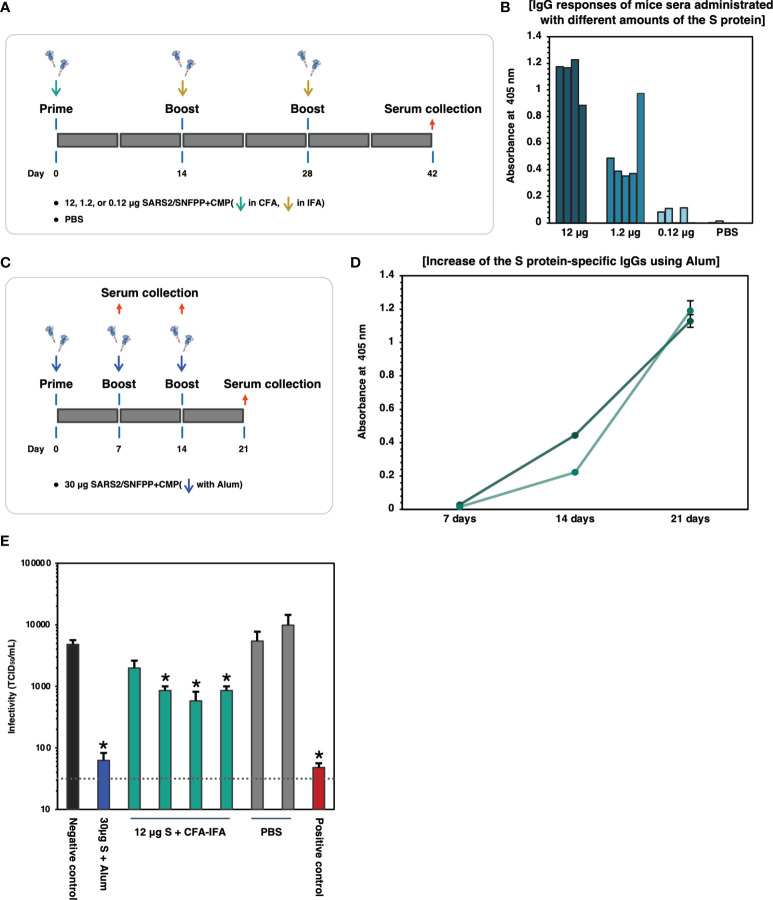
Binding and neutralizing activity of serum antibody in the mice immunized SARS-CoV-2 S protein. **(A)** Schematic representation of the immunization schedule of mice. BALB/c mice were inoculated 0.12, 1.2, and 12 µg of the antigens and boosted at day 14 and 28, respectively. CFA was used for the prime vaccination and IFA was used for the boost vaccinations. Sera were collected at 42 days after prime vaccination. **(B)** Protein antigen binding of IgG at the 1:1000 dilutions of serum from the immunized mice. The data shown represent OD 405 nm values of the individual mouse. **(C)** Schematic representation of the immunization schedule of mice using Alum adjuvant. BALB/c mice (n=2) were inoculated with 30 µg of the S protein and boosted at days 7 and 14, respectively. Sera were collected at 7, 14, 21 days after prime vaccination. **(D)** ELISA analysis to detect the increase of the S protein-specific IgGs in vaccinated mice sera (n=2). The sera were collected at each time point and used at 1:500 dilutions. Each with triplicate wells shows the data as mean ± SEM (OD 405 nm). **(E)** Neutralizing activity of immunized mice sera against SARS-CoV-2. 30µg S + Alum, mouse serum vaccinated with 30µg S protein in the presence of Alum adjuvant; 12µg S + CFA-IFA, mice sera vaccinated with 12µg S protein in the presence of CFA (prime) and IFA (boost); PBS, PBS inoculated mice sera. The serum from a COVID-19 patient was used as a positive control. The neutralizing activity of each serum was indicated as mean TCID_50_/mL ± SEM (n = 3). Statistical significance was tested by comparing the results with a negative control in an unpaired t-test (*P value < 0.05). The dashed line indicates the limit of detection.

SARS2/SNFPP+CMP+TEV-H8STREPH6 was predicted to be a promising candidate vaccine antigen because it is highly glycosylated and forms an appropriate structure that can bind to soluble hACE2 protein. Therefore, we evaluated the immunogenicity of the S protein in mice. First, 12, 1.2, and 0.12 μg of S proteins were administered to mice, respectively, and the induction of S protein-specific antibodies was measured. The S proteins were intraperitoneally administrated 3 times of 7-week-old Balb/c mice at 2-week intervals, using potent Complete Freund’s adjuvant (CFA) for prime vaccination and Incomplete Freund’s adjuvant (IFA) for boosting (**Figure 5A**). Serum was collected 2 weeks after the third inoculation and analyzed for S protein-specific IgG induction by ELISA. When each mouse serum was diluted 1000-fold, and IgG levels were measured on S-protein-coated ELISA plates, specific antibodies were detected in an inoculum dose-dependent manner (**Figure 5B**). Since CFA and IFA cannot be used as adjuvants for humans, we investigated the effect of aluminum hydroxide (Alum) adjuvant, which is commonly used for human vaccines. Alum adjuvant may have a lower capability to induce antibody production compared to the oil-based adjuvant, depending on the antigen administered (34, 35). Therefore, the dose of S protein was increased to 30 μg/dose, and as a preliminary experiment, two mice were boosted twice at weekly intervals, and serum was collected over time (**Figure 5C**). The results of the measurement of S protein-specific antibodies in mouse serum showed that the amount of antibodies increased over time, even when Alum adjuvant was used (**Figure 5D**). Then, these sera from immunized mice were used to examine neutralizing activity against SARS-CoV-2 infection. As an infection model, we used VeroE6/TMPRSS2 cell line that is highly susceptible to SARS-CoV-2 infection by constitutively expressing TMPRSS2, which is a host protease enhancing SARS-CoV-2 entry (36). Live SARS-CoV-2 was pre-incubated with each mouse serum from experimental groups indicated and challenged to VeroE6/TMPRSS2 cells, and TCID_50_ was calculated from the cytopathic effect in each well. As shown in **Figure 5E**, individual sera from mice immunized with 12 μg of S protein with CFA & IFA (4 mice, **Figure 5A**) and mixed sera from mice immunized with 30 μg of S protein with Alum (2 mice, **Figure 5C**) were used in the neutralization assay. Contrary to our initial expectation, sera from mice immunized with Alum showed potent inhibition of infection, comparable to a serum from a COVID-19 patient with strong neutralizing activity, while sera immunized with CFA&IFA showed weak activity (**Figure 5E**).

### Alum Adjuvant Is Effective in Inducing Neutralizing Antibodies to Suppress SARS-CoV-2 Infection Using the S-Subunit Vaccine

**Figure 6:**
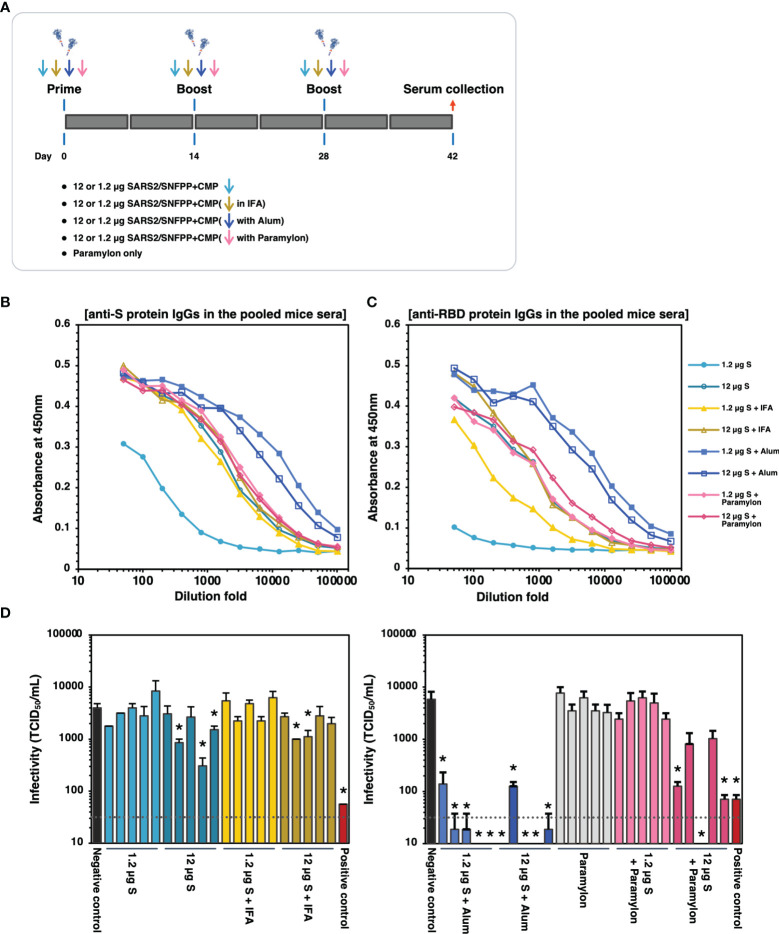
The effects of adjuvants inoculated with the S protein in mice. **(A)** Schematic representation of the immunization schedule of mice. BALB/c mice were inoculated with 1.2 and 12 µg of the antigens with IFA, Alum, or paramylon and boosted at days 14 and 28, respectively. Only paramylon was also inoculated (Paramylon only group). Sera were collected at 42 days after prime vaccination. **(B)** The IgG antibody level against S protein in vaccinated mice sera. Serial dilutions of pooled sera from each immunization group were used for ELISA analysis. **(C)** The IgG antibody level against RBD protein in vaccinated mice sera. Serial dilutions of pooled sera from each immunization group were used for ELISA analysis. **(D)** Neutralizing activity of immunized mice sera. Mouse serum inoculated with 30 μg of S protein in the presence of Alum adjuvant, which showed high neutralizing activity in **Figure 5E**, was used as a positive control. The neutralizing activity of each serum was indicated as mean TCID_50_/mL ± SEM (n = 3). Statistical significance was tested by comparing the results with a negative control in an unpaired t-test (*P value < 0.05). The dashed line indicates the limit of detection.

The trimeric protein of SARS2/SNFPP+CMP+TEV-H8STREPH6 from silkworm was suggested to induce potent neutralizing antibodies against SARS-CoV-2 in vaccinated mice using Alum adjuvant. To confirm this again and further test the effect of different adjuvants on humoral immunity, we increased the number of mice used and performed additional immunization experiments with IFA, Alum, and paramylon adjuvants (**Figure 6A**). All vaccinations were performed under the same conditions in this analysis, and serum antibodies binding to the S protein were measured by ELISA 42 days after the first administration. In the pooled sera of each inoculation group, the Alum adjuvant groups showed relatively higher antibody titers than the other adjuvant groups, even at a dose of 1.2 µg S protein (**Figure 6B**). The group using IFA or paramylon as adjuvant showed comparable antibody titer regardless of the S protein dose (**Figure 6B**). Without adjuvant, the administration of 12 µg S protein resulted in antibody titers comparable to those of IFA and paramylon, but the 1.2 µg S protein administration group showed lower antibody titers in serum than those with adjuvant (**Figure 6B**). Furthermore, when the antibody titers of the sera of individual mice were analyzed by ELISA, the results obtained were generally consistent with those of the pooled sera (**Supplementary Figure 2**). However, in the paramylon group, the difference in antibody levels among individuals was greater in the group inoculated with 1.2 µg of S protein than in the group inoculated with 12 µg of S protein (**Supplementary Figures 2H, I**). On the other hand, in ELISA with receptor binding domain (RBD) coated plates, specific antibody induction was more pronounced with Alum adjuvant, and dose-dependent antibody induction was observed with paramylon and IFA adjuvant (**Figure 6C** and **Supplementary Figure 3**). Interestingly, in the group inoculated with 12 µg of S protein, paramylon induced more specific antibodies than IFA (**Figure 6C**). This difference was more pronounced in the ELISA of individual sera (**Supplementary Figures 3D, I**). In the neutralization assay using VeroE6/TMPRSS2 cells, similar to the results of antibody titration, only samples from the Alum adjuvant group showed high neutralization activity even with a dose of 1.2 µg S protein, and some sera completely blocked SARS-CoV-2 infection (**Figure 6D**). In the group inoculated with 12 μg of S protein in paramylon, although there was no difference in the induced antibody titer, but some sera strongly suppressed viral infection (**Figure 6D**). On the other hand, the overall suppression was low in the groups inoculated with IFA or without an adjuvant except for some individuals showing statistically significant suppression of infection (**Figure 6D**). These results showed that a high amount of antibodies against RBD in immune sera coincided with high neutralizing activity against SARS-CoV-2. Collectively, these results indicate that SARS2/SNFPP+CMP+TEV-H8STREPH6 protein is an antigen that can strongly induce neutralizing antibodies and may provide sufficient protection from viral infection, especially in combination with Alum adjuvant.

## Discussion

More than 100 million people have been infected with the newly emerged SARS-CoV-2 in the world. For COVID-19 prevention, mRNA vaccines developed by Pfizer and BioNTech have already been approved and administrated, and clinical trials of several other vaccine formulations are under proceeding. However, there is still a need for safer vaccines that require smaller doses. In this study, to develop a low-cost subunit vaccine using the silkworm, the secretion efficiency was improved by fusing an appropriate heterotrimeric motif and affinity tag to the C-terminus of the ectodomain of the SARS-CoV-2 spike protein. Also, we have screened an inbred strain library of silkworms maintained by Kyushu University to find specific strains capable of high expression of S protein. S protein purified from silkworm larvae induced high levels of effective humoral immunity to SARS-CoV-2 when administered with appropriate adjuvants.

Using the silkworm-baculovirus expression vector system (silkworm-BEVS), we have succeeded in the mass-production of many recombinant proteins that are difficult to be expressed and secreted by other expression systems (21, 37). In addition, when BEVS is used, the silkworm host system often shows higher expression levels of recombinant proteins than the insect cultured cell host system. In this study, about 200 µg of purified SARS-CoV-2 S protein were obtained from 10 mL of silkworm larval serum. During the purification process, about 70% of the protein was lost because two-step affinity chromatography was employed to obtain a purified protein with high purity. Considering that about 0.2 to 0.4 mL of serum can be obtained from one silkworm larva, about 25 to 50 silkworm larvae are required to obtain 10 mL of serum. Since the silkworm is the only domesticated insect among arthropods and has been used for silk production for about 5,000 years, a rearing method that does not require complicated equipment has already been established (16). Furthermore, since about 1000 silkworms can be kept in a rearing cage of about 1m x 1m, it is easy to expand the rearing scale. In addition, this expression system uses an inbred silkworm strains that has been maintained at Kyushu University for more than 100 years. These silkworm strains are genetically homogenized, and therefore, inter-individual differences in the productivity of recombinant proteins within the same strain are less likely to occur. This contributes to maintaining the quality of recombinant protein antigens in silkworm-based vaccine production. Therefore, silkworms are expected to be a bioreactor that can reduce the cost of vaccine production.

Trimerization of viral surface glycoproteins is often a critical process for their proper folding, post-translational modifications (PTMs), and functionality for viral entry. Glycoproteins of many viruses, such as Coronaviruses and Retroviruses, also form trimeric structures on the surface of viral particles. In order to reproduce these trimerized antigens as recombinant proteins, these viral proteins were fused with foreign trimerization motifs such as GCN4, CMP, and T4 fibritin to improve solubility and stability through spontaneous trimer formation (38–43). In the paper that first reported the cryo-EM structure of the S protein of SARS-CoV-2, T4 fibritin was used to stabilize the structure of the S protein, replacing the furin cleavage site with GSAS and two specific amino acids in the HR1/CH domain with PP (**Figure 1B**) (5). Our previous paper showed that substitutions at furin cleavage sites increased secretion efficiency into silkworm serum, possibly because these substitutions prevented cleavage of the protein by silkworm proprotein convertases (20). In the present study, in addition to these substitutions, three trimerization motifs (GCN4, CMP, and T4 fibritin) were fused to the exodomain of the SARS-CoV-2 S protein, respectively. Among them, the S protein fused with T4 fibritin showed slightly higher expression than the other trimerization motifs, and CMP was the second most highly expressed (**Figure 3B**). CMP is an extracellular matrix protein abundant in many organisms, and the amino acid sequence is relatively conserved among them. The trimerization motif of CMP used in this study was derived from chicken, and its homology was 60.47% compared to the same region of human CMP (**Supplementary Figure 4**). For human vaccines, the human CMP would be expected to prevent its own immunogenicity, unlike the highly immunogenic T4 fibritin and GCN4, and would not interfere with the immune response to the vaccines (44, 45). Furthermore, CMP is the only one among the three motifs mentioned above with structural stability due to intermolecular disulfide bonds (46, 47). Therefore, the contribution of these motifs to the solubility and stability of the spike proteins might be different. Such factors need to be analyzed in more detail because they affect the shelf life of the S protein, its stability in the body, and its immune effect.

In many articles, SARS-CoV-2 S protein or its derivatives could be expressed successfully in mammalian cells (22, 30, 48). Although mammalian cells can produce recombinant proteins that are more appropriately folded and processed by PTMs in several cases, there is a risk of contamination of infectious microorganisms. Alternatively, the *E. coli* expression system is a robust tool for the mass production of recombinant proteins. However, there is no report of expression of the S protein of SARS-CoV-2 in *E. coli*, suggesting that the large and complicated structure of the spike protein is difficult to produce in prokaryotes. Although the glycans attached to the S protein are different between insects and mammals (49), the spike protein produced in the silkworm was mainly modified with high-mannose N-glycans and bound to the ACE2 receptor *in vitro*. It also elicited neutralizing antibodies in mice, indicating that it forms an appropriate structure that mimics the native S protein and has excellent quality as a subunit vaccine.

Many companies and researchers have accelerated the development of various vaccine candidates against SARS-CoV-2 (50), and recombinant protein vaccines produced by heterologous expression systems are a proven formulation. In SARS-CoV-2, RBD, a region essential for viral infectivity, is the primary target of neutralizing antibodies against the viral S protein (51, 52). Our validation of the adjuvant effect showed that the high neutralizing activity of mouse sera inoculated with the S protein against SARS-CoV-2 was consistent with the high IgG levels against RBD rather than that against the entire S ectodomain (**Figures 6C, D**). In addition, immunological studies of a protein subunit vaccine candidate using the RBD showed that it elicits neutralizing antibodies without toxicity in non-human primates (48). Recently, it has been reported that neutralizing epitopes are also present in several regions of the S protein other than the RBD, such as the N-terminal domain (51, 53, 54). Other studies have shown that inoculation with the S protein induces higher levels of humoral immunity with neutralizing antibodies than inoculation with the RBD, indicating that the more immunogenic S protein serves as a more reliable vaccine antigen (55).

In immunological experiments in mice, antigen-specific antibody responses were observed in all groups inoculated with S antigen, but in subsequent neutralization tests, only the group adjuvanted with Alum effectively neutralized SARS-CoV-2. The insufficient immunogenicity of antigen protein alone is one of the major drawbacks of subunit vaccines, which is consistent with the present results. However, in addition to this result, a potent incomplete Freund’s adjuvant (IFA), which is commonly used in immunization studies of animals such as mice, also failed to block viral infection (**Figure 6D**). A study using the S protein produced by cultured insect cell-baculovirus expression vector system has reported that the use of complete Freund’s adjuvant (CFA) for S protein inoculation (three times administration) produced neutralizing antibodies against SARS-CoV-2 in mice that were comparable to those produced by alum adjuvant (14). In that report, 35 µg of trimerized S protein with furin cleavage site and without two proline mutations was administered to mice, so the physicochemical properties of antigen protein by mixing with adjuvant may be different, which possibly be the reason for the difference from our results. In addition, in our initial immunization experiments, CFA was used only for the prime vaccination and IFA was used for the two subsequent boosting, but the neutralizing activity of SARS-CoV-2 in mouse serum was weak (**Figures 5A, E**). The difference between CFA and IFA is the presence or absence of killed mycobacteria, and CFA containing microbial components may contribute to the induction of protective immunity during boost vaccination. In a study comparing the effects of several adjuvants, including the oil emulsion adjuvant AS03, on the immune effects of RBD nanoparticles in non-human primates, squalene-based AS03 and Alum strongly induced neutralizing antibodies (56). On the other hand, the squalene-based oil-in-water (O/W) adjuvant, which is the same as AS03, induced less neutralizing antibodies, suggesting that other components may have different effects on immune induction even though the main components are the same. Similar to AS03, another squalene-based adjuvant, MF59, has been used in clinical studies human SARS-CoV-2 S ectodomain as an antigen, and has been shown to safely induce a neutralizing immune response (57). Therefore, in future studies, the effects of such oil-emulsion type adjuvants that can be used in humans should be compared in animal experiments using the S antigens produced by silkworm-BEVS.

In this study, we demonstrated for the first time the efficacy of paramylon as an adjuvant in a subunit vaccine formulation of SARS-CoV-2. Paramylon, a storage polysaccharide of the microalga *Euglena gracilis*, is a β-glucan that is recognized as pathogen-associated molecular patterns (PAMPs) by the pattern recognition receptors (PRRs) of cells responsible for innate immunity (58, 59). Such an effect might contribute to the induction of stronger neutralizing antibodies than IFA, and inoculation with 12 µg of S antigen showed a superior protective effect. However, even though there was no difference in the amount of S or RBD protein-specific IgG, the strength of neutralizing activity varied greatly among individual mice, raising concerns about the stability to neutralizing antibody induction. The variability among individuals may be due to the fact that β-glucan interacts with the S protein, causing a conformational change. It may be practical to use paramylon in combination with other effective adjuvants such as Alum to reduce the dosage of the S protein or target additional immune effects.

On the other hand, the group that achieved the most protective immune induction in this study was the group treated with Alum adjuvant. Aluminum salt-based adjuvants are widely used in approved vaccines for humans, including some protein-based subunit vaccines, such as virus-like particles of human papillomavirus. The mechanism of the Alum adjuvant effect has long been thought to be the promotion of antigen depot in the inoculated body, but more recently, it has been suggested that it also induces a strong immune response by recruitment of innate immune cells and activating innate immunity or antigen-presentation to APCs (60, 61). Stimulation of innate immunity, in addition to stimulation by antigens, is crucial for the activation of antigen-presenting cells for efficient induction of acquired immunity (62). Therefore, the differences in antibody induction by different adjuvants in this study might reflect the type of innate immune pathway stimulated by each adjuvant and the intensities of the stimulations (63). In another study, immunological analysis with S-protein produced by 293-F cells using several adjuvants showed that neutralizing antibodies were induced after a single administration (64). Still, the kinetics of antibody elicitation were different, and the cytokine profiles were also other. In the case of Alum, IL-10 and IL-5, which are mainly produced by Th2 cells, were induced.

The results of immunization experiments in mice or non-human primates shown in several studies, including this study, warrant the contribution of Alum adjuvants in making subunit vaccines of SARS-CoV-2 more efficacious (14, 65). However, in immunological studies of SARS-CoV vaccines, the fact that Alum induces a Th2-biased immune response, leading to an increase in eosinophils and inflammatory infiltrates by viral challenge, has been raised as a safety risk (66, 67). To respond rapidly against widespread infectious diseases, it is imperative to use the immunogenicity of existing formulations to create safe and efficient vaccines. The most familiar adjuvants are Aluminum hydroxide (Alum) is the only adjuvant used in most countries. Therefore, research on combinations of Alum with other adjuvants and improved versions of Alum formulation is ongoing. For example, the combination of CpG and Alum or other adjuvants or Alum emulsified with squalene to induce balanced Th1/Th2 or Th1-biased immune responses is a preferred strategy for future subunit vaccine formulation (56, 65, 68–70).

In conclusion, we constructed the trimerized spike protein of SARS-CoV-2 using CMP as a trimerization domain. The trimeric spike protein fused with the purified tag was secreted normally from the serum of silkworm larvae and was easy to purify. Also, specific silkworm strains showed relatively higher spike protein expression. In the future, the recovery of SARS-CoV-2 S protein from one silkworm larva is expected to be improved more than five-fold by validating the recovery process in detail during the purification and by utilizing hybrid vigor in F1 lines crossed with more effective silkworm strains. Trimerized S protein mass-produced in silkworm induced a very strong SARS-CoV-2 neutralizing activity in the sera of immunogen-inoculated mice by adding Alum adjuvant. Although further clinical experiments are needed to confirm the efficacy against SARS-CoV-2, our results indicated that the recombinant trimerized spike protein expressed in silkworm might be a promising vaccine candidate preventing COVID-19.

## Data Availability Statement

The original contributions presented in the study are included in the article/**Supplementary Material**. Further inquiries can be directed to the corresponding author.

## Ethics Statement

The animal study was reviewed and approved by “Institutional Animal Care and Use Committee of Kyushu University” and “Animal Care and Use Committee of Kagoshima University”.

## Author Contributions

AM designed and operated the experiments and drafted the manuscript. JL designed expression constructs and generated recombinant baculoviruses. TMi managed mouse vaccination, blood collection, and ELISA analysis, and analyzed data from immunization experiments. HM designed and constructed plasmid vectors. KSa and KO managed mouse vaccinations, blood collections, and ELISA analysis. YS and JY performed SARS-CoV-2 neutralization assays. DT and TU designed and supervised animal experiments. YK and MN conducted preliminary experiments to analyze the neutralizing activity of vaccinated mice sera. NK, KK, TE, TN, and MH performed protein purifications and quantifications. AN and KSu prepared and provided paramylon from *Euglena gracilis*. YT, MT, TMo, and HN contributed to rearing silkworms, baculovirus infection, and serum collections. RF analyzed the data and provided ideas. TK conceived this study, provided ideas, and revised the manuscript. All authors contributed to the article and approved the submitted version.

## Funding

This work was supported by MEXT (Ministry of Education, Culture, Sports, Science, and Technology) Grant, Kyushu University operating expenses, and under the “COVID-19 Drug and Vaccine Development Donation Account” Project from Sumitomo Mitsui Trust Bank.

## Conflict of Interest

AN and KSu are employed by euglena Co., Ltd. HN is employed by KAICO Ltd.

The remaining authors declare that the research was conducted in the absence of any commercial or financial relationships that could be construed as a potential conflict of interest.

## Publisher’s Note

All claims expressed in this article are solely those of the authors and do not necessarily represent those of their affiliated organizations, or those of the publisher, the editors and the reviewers. Any product that may be evaluated in this article, or claim that may be made by its manufacturer, is not guaranteed or endorsed by the publisher.
