# Supplementary material for: Optimization of SARS-CoV-2 Spike Protein Expression in the Silkworm and Induction of Efficient Protective Immunity by Inoculation With Alum Adjuvants
Source: Front Immunol. 2022 Jan 12;12:803647. doi: 10.3389/fimmu.2021.803647 (PMC8789674; doi:10.3389/fimmu.2021.803647)
Supplement: Supplementary file 1 [file DataSheet_1.pdf]

Supplementary Material

Supplementary Figures

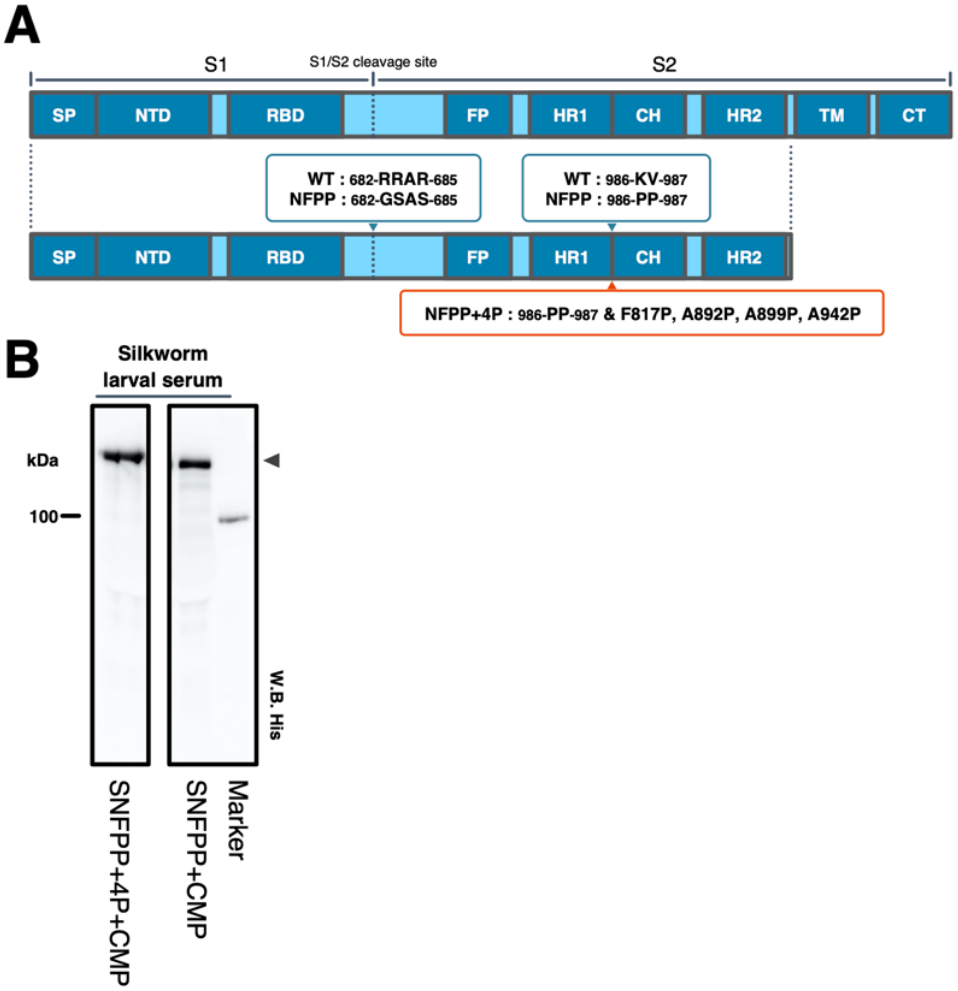

Supplementary Figure 1. Expression analysis of SARS-CoV-2 SNFPP protein with additional four proline substitutions (NFPP+4P). **(A)** A map of the domains of the spike protein. SP, signal peptide; NTD, N-terminal domain; RBD, receptor binding domain; FP, fusion peptide; HR1, heptad repeat 1; CH, central helix; HR2, heptad repeat 2. The substitution sites of proline were indicated in the red square. **(B)** Expression of SARS-CoV-2 spike protein in silkworm larva. The pooled serum of silkworm larvae expressing SNFPP+4P+CMP or SNFPP+CMP were collected at 4 days after recombinant BmNPV infection. All samples (sera of 1μL) were analyzed by SDS-PAGE followed by western blotting using HisProbe-HRP. Arrowhead indicates the position of the S protein. Molecular sizes (kDa) of the protein marker are indicated on the left.

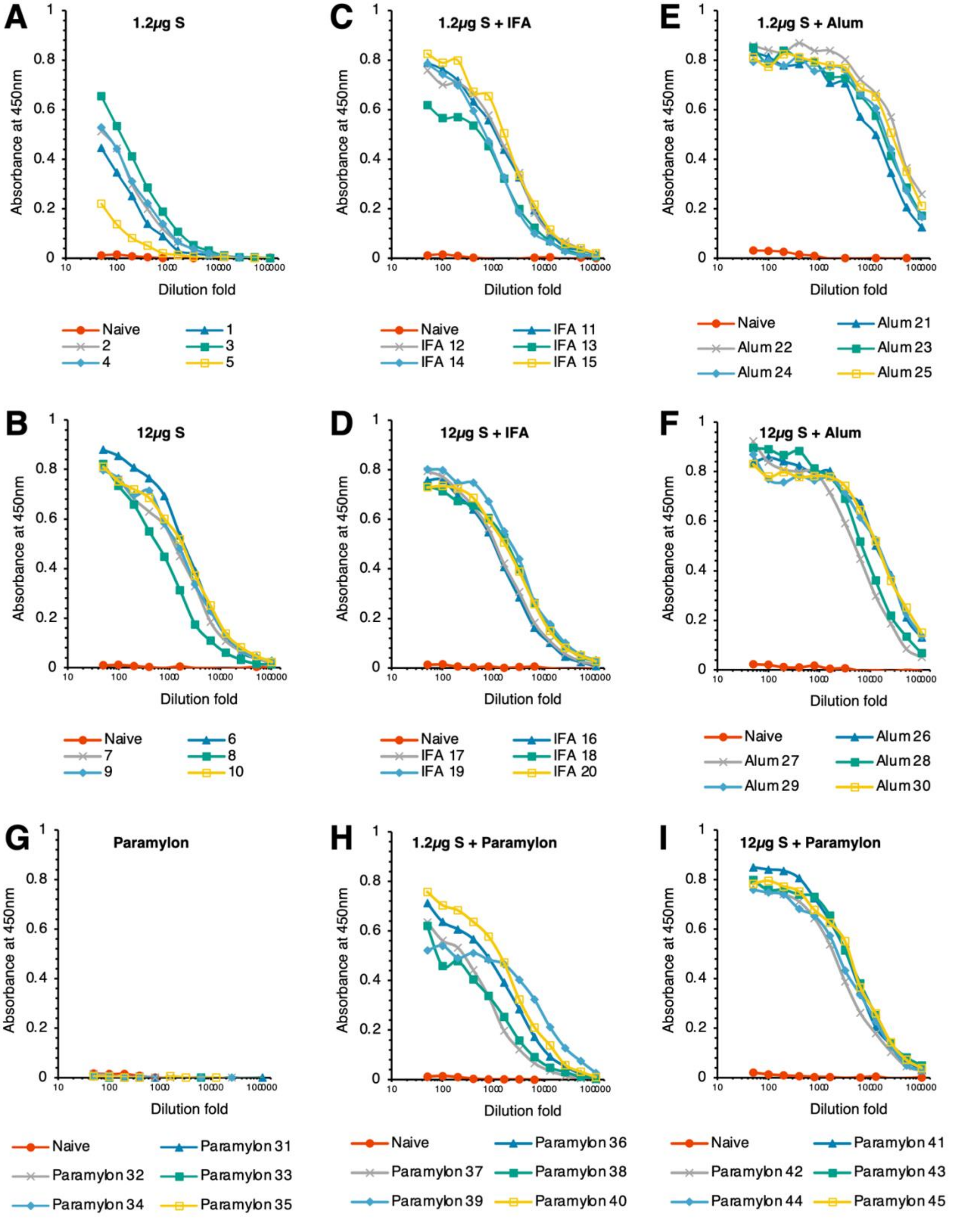

Supplementary Figure 2. ELISA titrations for SARS-CoV-2 S protein-binding IgG antibodies in the sera of individual mice. The SARS2/SNFPP+CMP+TEV-H8STREPH6 protein was used for coating

the 96-well plates. Mice sera inoculated with 1.2 or 12 µg S protein without adjuvant **(A, B)**, using incomplete Freund's adjuvant (IFA) **(C, D)**, or using Alum adjuvant **(E, F)**. **(G)** Mice sera inoculated with only Paramylon. **(H, I)** Mice sera inoculated with 1.2 or 12 µg S protein using Paramylon. Each serum was used in serial dilutions from 1:50 to 1:102400. As a negative control, the serum from naïve mouse was used.

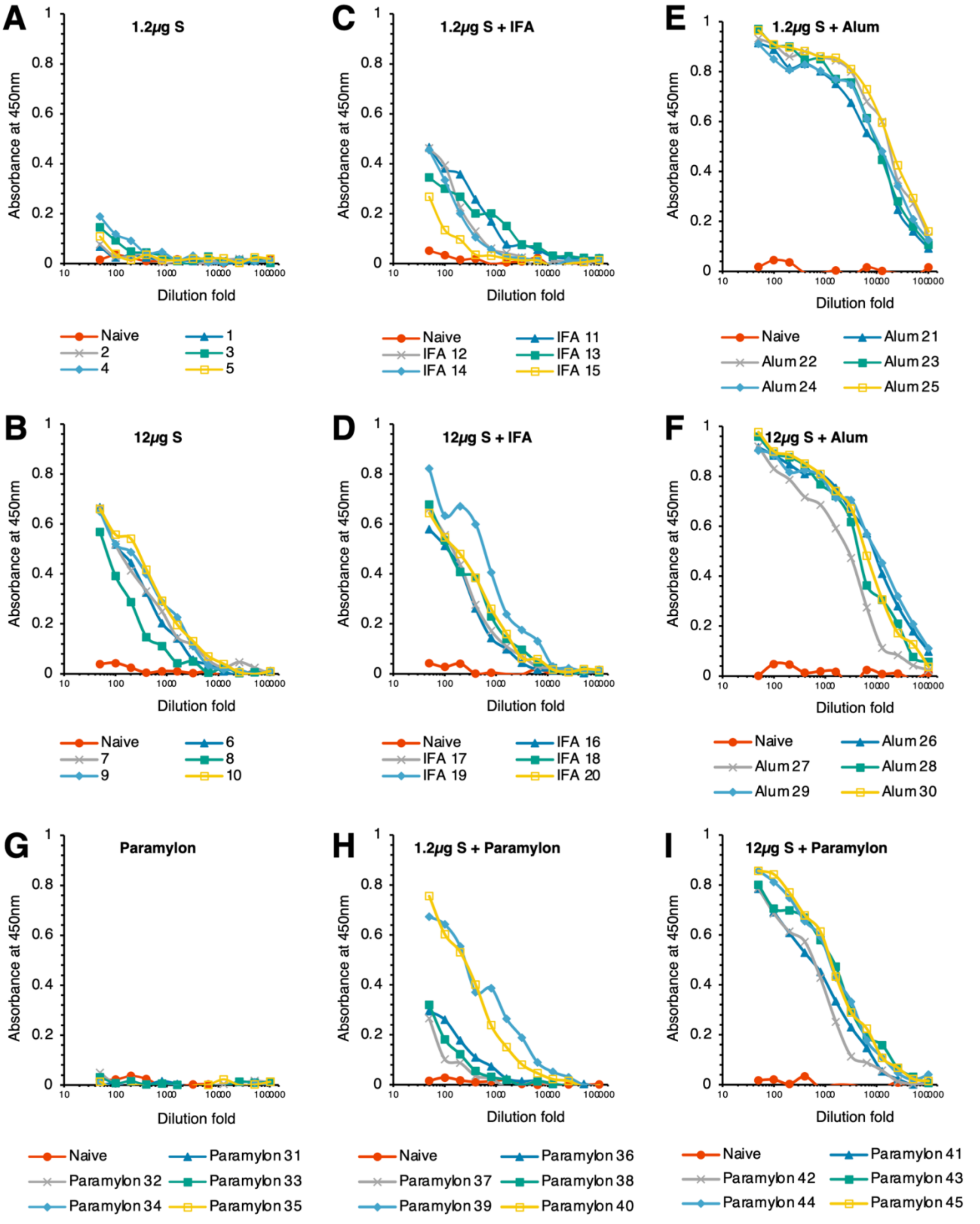

Supplementary Figure 3. ELISA titrations for SARS-CoV-2 RBD protein-binding IgG antibodies in the sera of individual mice. The SARS-CoV-2 SUMO-RBD+TEV-H8STREPH6 protein was

produced using silkworm-baculovirus expression vector system and purified from silkworm serum using the two-step chromatography as well as the recombinant S proteins. Mice sera inoculated with 1.2 or 12 µg S protein without adjuvant **(A, B)**, using incomplete Freund's adjuvant (IFA) **(C, D)**, or using Alum adjuvant **(E, F)**. **(G)** Mice sera inoculated with only Paramylon. **(H, I)** Mice sera inoculated with 1.2 or 12 µg S protein using Paramylon. Each serum was used in serial dilutions from 1:50 to 1:102400. As a negative control, the serum from naïve mouse was used.

```
CLUSTAL O(1.2.4) multiple sequence alignment
SP|P21941|MATN1_HUMAN MRVLSGTSLMLCSLLLLL----QALCSPGLAPQSRGHLCRTRPTDLVFVVDSSRSVRPVE 56
SP|P51942|MATN1_MOUSE MKVTSGPAFALCSLLLLLLLLLQVPDSLSLVPQPRGHLCRTRPTDLVFVVDSSRSVRPVE 60
TR|I3L5Q7|I3L5Q7_PIG  MRVTCGPSPVLCGLLLLL----QAPHTLGLAPLSRGPLCRTRPTDLVFVVDSSRSVRPVE 56
SP|P05099|MATN1_CHICK ------MDGIFCALPLSLLLLLQSCGVWGAPPQPRGTLCRTKPTDLVFIIDSSRSVRPQE 54
                                :*.* * *    *     .  *  ** ****:******::******** *

SP|P21941|MATN1_HUMAN FEKVKVFLSQVIESLDVGPNATRVGMVNYASTVKQEFSLRAHVSKAALLQAVRRIQPLST 116
SP|P51942|MATN1_MOUSE FEKVKVFLSQVIESLDVGPNATRVGLVNYASTVKPEFPLRAHGSKASLLQAVRRIQPLST 120
TR|I3L5Q7|I3L5Q7_PIG  FEKVKVFLSQVIESLDVGPNATRVGLVNYASSVKQEFPLRAHSSKAALLQAVRRIQPLST 116
SP|P05099|MATN1_CHICK FEKVKVFLSRVIEGLDVGPNSTRVGVINYASAVKNEFSLKTHQTKAELLQAVQRIEPLST 114
                      *********:***.******:****::****:** ** *::* :** *****:**:****

SP|P21941|MATN1_HUMAN GTMTGLAIQFAITKAFGDAEGGRSRSPDISKVVIVVTDGRPQDSVQDVSARARASGVELF 176
SP|P51942|MATN1_MOUSE GTMTGLALQFAITKALSDAEGGRARSPDISKVVIVVTDGRPQDSVRDVSERARASGIELF 180
TR|I3L5Q7|I3L5Q7_PIG  GTMTGLAIHFAITKALSDAEGGRPRSPDISKVVIVVTDGRPQDSVRDVSARARASGIELF 176
SP|P05099|MATN1_CHICK GTMTGLAIQFAISRAFSDTEGARLRSPNINKVAIVVTDGRPQDGVQDVSARARQAGIEIF 174
                      *******::***::*:.*:**.* ***:*.**.**********.*:*** *** :*:*:*

SP|P21941|MATN1_HUMAN AIGVGSVDKATLRQIASEPQDEHVDYVESYSVIEKLSRKFQEAFCVVSDLCATGDHDCEQ 236
SP|P51942|MATN1_MOUSE AIGVGRVDKATLRQIASEPQDEHVDYVESYNVIEKLAKKFQEAFCVVSDLCATGDHDCEQ 240
TR|I3L5Q7|I3L5Q7_PIG  AIGVGRVDKATLQQIASEPQDEHVDYVESYSVIEKLSKKFQEAFCLVSDLCATGDHDCEQ 236
SP|P05099|MATN1_CHICK AIGVGRVDMHTLRQIASEPLDDHVDYVESYSVIEKLTHKFQEAFCVVSDLCATGDHDCEQ 234
                      ***** **  **:****** *:********.*****::*******:**************

SP|P21941|MATN1_HUMAN VCISSPGSYTCACHEGFTLNSDGKTCNVCSGGGGSSATDLVFLIDGSKSVRPENFELVKK 296
SP|P51942|MATN1_MOUSE LCVSSPGSYTCACHEGFTLNSDGKTCNVCRGGGSGSATDLVFLIDGSKSVRPENFELVKK 300
TR|I3L5Q7|I3L5Q7_PIG  VCLSSPGSYTCACREGFTLNSDGKTCNVCSGGGGSLATDLVFLIDGSKSVRPENFELVKK 296
SP|P05099|MATN1_CHICK ICISTPGSYKCACKEGFTLNNDGKTCSACSG-GSGSALDLVFLIDGSKSVRPENFELVKK 293
                      :*:*:****.***:******.*****..* * *.. * **********************

SP|P21941|MATN1_HUMAN FISQIVDTLDVSDKLAQVGLVQYSSSVRQEFPLGRFHTKKDIKAAVRNMSYMEKGTMTGA 356
SP|P51942|MATN1_MOUSE FINQIVDTLDVSDRLAQVGLVQYSSSIRQEFPLGRFHTKKDIKAAVRNMSYMEKGTMTGA 360
TR|I3L5Q7|I3L5Q7_PIG  FINQIVDTLDVSDKLAQVGLVQYSSSVRQEFPLGRFHTKKDIKAAVRNMSYMEKGTMTGA 356
SP|P05099|MATN1_CHICK FINQIVESLEVSEKQAQVGLVQYSSSVRQEFPLGQFKNKKDIKAAVKKMAYMEKGTMTGQ 353
                      **.***::*:**:: ***********:*******:*:.********::*:*********

SP|P21941|MATN1_HUMAN ALKYLIDNSFTVSSGARPGAQKVGIVFTDGRSQDYINDAAKKAKDLGFKMFAVGVGNAVE 416
SP|P51942|MATN1_MOUSE ALKYLIDNSFTVSSGARPGAQKVGIVFTDGRSQDYINDAARKAKDLGFKMFAVGVGNAVE 420
TR|I3L5Q7|I3L5Q7_PIG  ALKYLIDNSFTVSSGARPGAQKVGVVFTDGRSQDYINDAAKKAKDLGFKMFAVGVGNAVE 416
SP|P05099|MATN1_CHICK ALKYLVDSSFSIANGARPGVPKVGIVFTDGRSQDYITDAAKKAKDLGFRMFAVGVGNAVE 413
                      *****:*.**:::.*****. ***:***********.***:*******:***********

SP|P21941|MATN1_HUMAN DELREIASEPVAEHYFYTADFKTINQIGKKLQKKICVEEDPCACESLVKFQAKVEGLLQA 476
SP|P51942|MATN1_MOUSE EELREIASEPVADHYFYTADFKTINQIGKKLQKQICVEEDPCACESILKFEAKVEGLLQA 480
TR|I3L5Q7|I3L5Q7_PIG  DELREIASEPVAEHYFYTADYKTINQIGKKLQKKICVEEDPCACESIVKFQSKVEGLLQA 476
SP|P05099|MATN1_CHICK DELREIASEPVAEHYFYTADFRTISNIGKKLQMKICVEEDPCECKSIVKFQTKVEELINT 473
                      :***********:*******::**.:****** :******** *:*::**::*** *:::

SP|P21941|MATN1_HUMAN LTRKLEAVSKRLAILENTVV 496
SP|P51942|MATN1_MOUSE LTRKLEAVSGRLAVLENRII 500
TR|I3L5Q7|I3L5Q7_PIG  LTRKLEAVSKRLAILENRIV 496
SP|P05099|MATN1_CHICK LQQKLEAVAKRIEALENKII 493
                      * :*****: *:  *** ::
```

Supplementary Figure 4. Sequence alignment of the full-length CMP from human (UniProt ID: P21941), mouse (UniProt ID: P51942), pig (UniProt ID: I3L5Q7), and chick (UniProt ID: P05099) using Clustal Omega (version 1.2.4). Conserved amino acids (asterisks) and semiconserved amino

acids (colons and periods) are indicated below the alignment. The trimerization motif of CMP used in this study was highlighted in yellow.
